# Chitosan-collagen-hydroxyapatite membranes for tissue engineering

**DOI:** 10.1007/s10856-022-06643-w

**Published:** 2022-01-24

**Authors:** José Becerra, Mariano Rodriguez, Dayana Leal, Karem Noris-Suarez, Gema Gonzalez

**Affiliations:** 1 Instituto de Ciencias Básicas, Universidad Técnica de Manabí, Portoviejo, Ecuador; 2 Lab. de Materiales, Centro de Ingeniería de Materiales y Nanotecnología, Instituto Venezolano de Investigaciones Científicas, IVIC, Caracas, Venezuela; 3 Lab. Ing. Tejidos, Universidad Simón Bolívar, Caracas, Venezuela; 4 Yachay Tech University, School of Physical Sciences and Nanotechnology, Urcuqui, 100119 Ecuador

## Abstract

Tissue engineering is growing in developing new technologies focused on providing effective solutions to degenerative pathologies that affect different types of connective tissues. The search for biocompatible, bioactive, biodegradable, and multifunctional materials has grown significantly in recent years. Chitosan, calcium phosphates collagen, and their combination as composite materials fulfill the required properties and could result in biostimulation for tissue regeneration. In the present work, the chitosan/collagen/hydroxyapatite membranes were prepared with different concentrations of collagen and hydroxyapatite. Cell adhesion was evaluated by MTS assay for two in vitro models. Additionally, cytotoxicity of the different membranes employing hemolysis of erythrocytes isolated from human blood was carried out. The structure of the membranes was analyzed by X-rays diffraction (XRD) and Fourier transform infrared spectroscopy (FTIR), scanning electron microscopy (SEM), and thermal stability properties by thermogravimetric methods (TGA). The highest cell adhesion after 48 h was obtained for chitosan membranes with the highest hydroxyapatite and collagen content. All composite membranes showed good cell adhesion and low cytotoxicity, suggesting that these materials have a significant potential to be used as biomaterials for tissue engineering.

**Figure:**
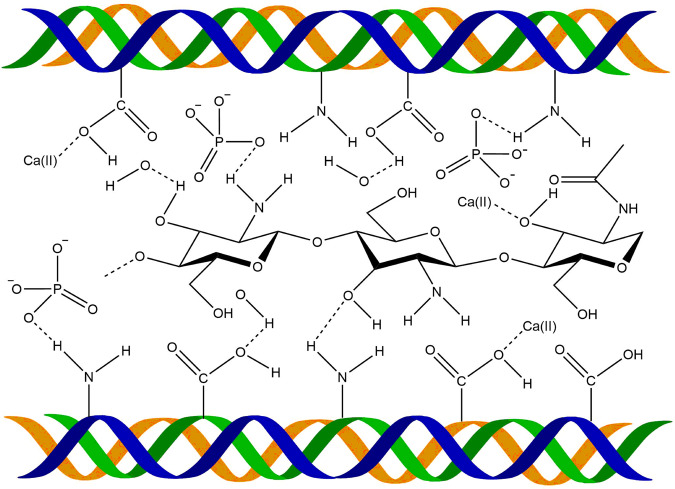
Graphical abstract

Graphical abstract

## Introduction

Tissue engineering is an area of great interest in the biomedical field that can regenerate different tissues: cartilage, epithelial tissue, and bone. In this context, tissue engineering searches to resolve the need to replace and regenerate damaged tissue. Therefore, several works seek materials to stimulate, guide, and improve diverse connective tissue regeneration, including skin, cartilage, and bone, with the least possible undesirable effect [1–5]. In this way, many ceramic and polymeric materials present a strong potential for tissue regeneration due to their biological, chemical, physical, and mechanical properties and their capacity to form composite materials with enhanced properties. Hydroxyapatite is very well known for its high biocompatibility [6] and its capacity to form complex biocomposites structures with enhancing bone regeneration properties [7, 8]. Additionally, it has been studied with different compounds for skin regeneration [9–11]. On the other hand, collagen (Col) is the most abundant protein in the extracellular matrix and the major structural element of all connective tissue. It is responsible for tissue stability and presents excellent biocompatibility [12–19]. The essential role of collagen in biomineralization has been reported [20]. Besides, chitosan looks promising to accomplish the necessary conditions for connective tissue healing and regeneration due to its excellent biological properties such as biocompatibility, biodegradability, and cytocompatibility [21–27]. Moreover, due to its excellent film-forming properties, many medical and pharmaceutics applications have been considered [28–32]. It can form complexes with other macromolecules and with diverse drugs and proteins [31–33], and it is compatible with other materials such as metals, polymers, and proteins to form bio-functional composite materials [6, 18, 19, 25, 34, 35]. It has been reported that chitosan-based materials can be used in cartilage tissue applications due to similarities found with glycosaminoglycans prompting the restoration of articular cartilage [36]. Chitosan/collagen scaffolds have been used for bone [37] and skin tissue engineering crosslink with glutaraldehyde to improve biostability [38, 39].

The properties of these materials open a wide range of possibilities in composite biomaterials considering tissue engineering and biomedical applications.

Thereby, different combinations of these materials have been carried out with promising results. In this way, hydroxyapatite-collagen scaffolds have been used very successfully in periodontal surgery, improving osteoinductivity and osteoconduction [40, 41]. On the other hand, chitosan/hydroxyapatite has been proved as a scaffold for bone tissue regeneration with promising results [24, 33, 40, 42–44]. The NH3 groups of chitosan interact with Ca and P ions of HA, providing the nucleation points in the structure [45]. Besides, the fluid retention and the degradation rate of the chitosan matrix in the scaffold vary by changing chitosan concentration or the proportion of Ca/P [45, 46]. In vivo studies in tibial bones of rats showed good osteoconductive properties with an almost entirely bone regeneration after 24 h [47]. More recently, Zhang performed In vivo tests in rabbits, resulting in complete bone regeneration after 16 weeks [48]. Also, hydrogels of chitosan/hydroxyapatite with good mechanical properties, cytocompatibility, and antimicrobial activity were developed for cartilage regeneration [49]. Chitosan/hydroxyapatite membranes have also been reported with good biomineralization results [50–55]. Membranes are of significant importance in biomedical applications; they can be used for bone regeneration (GBR) [56–58], drug delivery [59], coatings for implants [60], wound healing [61], and other vital applications on guided tissue regeneration.

Composites with combinations of chitosan/hydroxyapatite/collagen scaffolds with 3D interconnected porosity have been very well studied for tissue regeneration, reporting good biocompatibility, bone defects restoration, and low cytotoxicity [46, 62–66]. However, there are scarce reports on membranes formed with these three compounds. Teng et al. [67] prepared a sandwich structure membrane system with top and bottom layers made of collagen 20 wt% hydroxyapatite (HA) and chitosan in the middle layer. They reported good mechanical properties and good bioactivity.

Although chitosan/hydroxyapatite/collagen scaffolds have been studied with good results, works on chitosan/hydroxyapatite/collagen membranes lack more profound studies, and the effect of different compound concentrations on the properties of these materials has not been reported up to now, as far as the author’s knowledge.

Hence, taking advantage of the properties of each compound (chitosan, collagen, and hydroxyapatite) and the characteristics of good film-forming, good biocompatibility, and biodegradability, in the present work a detailed study of preparation and characterization of chitosan-based composite membranes, incorporating collagen and/or hydroxyapatite in different proportions (10 and 50% related to chitosan concentration) were prepared, for potential uses in tissue engineering. Biocompatibility and viability assays were carried out to determine if these materials could be used in bone or skin tissue engineering on future projects.

## Materials and methods

### Materials

Chitosan (Cs) (from Shrimp Shells, degree of deacetylation ≥75%) and collagen (Col) Type I, Insoluble (from Bovine Achilles Tendon) were purchased from Sigma-Aldrich. Hydroxyapatite (HA) was synthesized in our laboratory following the reaction:

$$10{{{\mathrm{Ca}}}}\left( {{{{\mathrm{OH}}}}} \right)_{2} \,+\, 6\left( {{{{\mathrm{NH}}}}_{4}} \right)2{{{\mathrm{HPO}}}}_{4} \to {{{\mathrm{Ca}}}}10\left( {{{{\mathrm{PO}}}}_{4}} \right)6\left( {{{{\mathrm{OH}}}}} \right)_{2} \,+\, 12{{{\mathrm{NH}}}}_{4}{{{\mathrm{OH}}}} + 6{{{\mathrm{H}}}}_{2}{{{\mathrm{O}}}}$$

Briefly, to a Ca(OH)2 suspension in deionized water, and ammonium phosphate solution was added dropwise. The solution was mixed under vigorously stirring at a constant temperature of 25 °C. The pH of this solution was adjusted to 10 and then left standing for 16 h at room temperature. The product was separated from the mother solution and successively washed until neutral pH. The powder obtained was dried at 60 °C for 20 h. The final material was characterized by X-rays Diffraction (XRD) and Fourier Transform Infrared Spectroscopy (FTIR).

### Chitosan composite membranes formation

#### Chitosan membranes

Chitosan membranes (Cs) were prepared by solvent casting method. Chitosan solution was obtained by dispersing 1.5% (w/v) in an aqueous solution of acetic acid at 2% (v/v). The solution was mechanically stirred until solubilization. After that, 25 g of solution was poured into Petri dishes (diameter = 8.2 cm) and dried at room temperature (~a week).

#### Chitosan/collagen composite membranes

Chitosan/Collagen composite membranes (Cs/Col) were obtained varying collagen content into the chitosan solution. Briefly, collagen (0.15 and 0.75% w/v) was dispersed into a chitosan solution prepared as described above by mechanical stirred until a good collagen dispersion was obtained. Then, 25 g of solution was poured into Petri dishes and dried at room temperature.

#### Chitosan/hydroxyapatite composite membranes

Chitosan/hydroxyapatite composite membranes (Cs/HA) were obtained varying HA content into the chitosan solution. Briefly, HA (0.15 and 0.75% w/v) was dispersed into a chitosan solution prepared as described above by mechanical stirring until a good dispersion of HA was obtained. Then, 25 g of solution was poured into Petri dishes (diameter = 8.2 cm) and dried at room temperature (~a week).

#### Chitosan/collagen/hydroxyapatite membranes

Chitosan/Collagen/Hydroxyapatite membranes (Cs/Col/HA) were obtained, varying HA and collagen content into the solution. Briefly, HA (0.15 and 0.75% w/v) and collagen (0.15 and 0.75% w/v) were dispersed into a chitosan solution prepared as described above by mechanical stirring until a good dispersion of HA and collagen was obtained. Then, 25 g of solution was poured into Petri dishes (diameter = 8.2 cm) and dried at room temperature (~a week).

The nomenclature used for the differently prepared samples is given in Table 1.

**Table 1:** Nomenclature of the materials prepared with different compositions

Bi-composite membranes	Chitosan (%wt/v in solution)	Collagen (%wt/v in solution)	Hydroxyapatite (%wt/v in solution)
BCC1	1.5	0.15	0
BCC2	1.5	0.75	0
BCHA3	1.5	0	0.15
BCHA4	1.5	0	0.75
Tri-composite membranes			
TC1	1.5	0.15	0.15
TC2	1.5	0.15	0.75
TC3	1.5	0.75	0.15
TC4	1.5	0.75	0.75

### Characterization of membranes

#### X-rays diffraction (XRD)

The microstructural characterization of all the prepared membranes was carried out by XRD, in a Siemens 5005 X-rays diffractometer using Cu Kα radiation (Ni filter) operating at 40 KeV and 20 mA in a range of 2θ = 5–90°.

#### FTIR analysis

To evaluate the possible interactions between the chitosan, hydroxyapatite, and collagen molecules, FTIR analysis was performed with a Perkin Elmer Spectrum100 equipment, using the Horizontal Attenuated Total Reflectance mode in the wavenumber range from 650 to 4000 cm^−1^.

#### Thermogravimetric analysis (TGA)

Thermogravimetric analysis was carried out by using a METTLER TOLEDO, TGA/DSC 1 STARe SYSTEM with a scan range from 25 to 600 °C, a heating rate of 10 °C per min^−1^, using air as reactive gas and nitrogen as protective gas, with a flux of 60 mL/min.

#### Isolation and expansion of human mesenchymal stromal cells from buccal fat pad (hBFP-MSCs)

Buccal fat pads (BFP) were obtained following a modified method of Farre-Guasch et al. [68]. Briefly, a BFP was obtained from healthy individuals undergoing elective orthognathic surgery procedures in Central University of Venezuela (UCV), Oral Surgery Department (Caracas, Venezuela) under informed consent of the donor and Ethics Committee of UCV approval.

Raw oral fat tissue was washed several times with sterile phosphate-buffered saline (PBS), minced into small pieces, and treated with 0.075% collagenase I (Sigma, St. Louis, MO) in α-MEM, for 60 min at 37 °C. After incubation, adipose tissue was centrifuged at 400 *g* for 10 min to separate the adipocytes and lipid droplets from the stromal vascular fraction (SVF).

SVF cells were resuspended in α-MEM medium containing 15% fetal bovine serum and 100 units/mL antibiotics/antimycotics solution. Suspended cells were passed through a 100 mm cell strainer (BD Biosciences, Palo Alto, CA), cells were counted, and their viability was assessed with Trypan Blue exclusion. Cells were seeded at 5 × 10^3^ cells/cm^2^ in 100 mm tissue culture dishes and maintained in a humidified incubator at 37 °C and 5% CO_2_.

#### Indirect cytotoxicity test

We approached a hemolysis test as a first step to determining these membranes’ biocompatibility because it is an easy and quick test to assess if these materials can be cytotoxic for other cell types [69].

Cytotoxicity of the different membranes was determined by the assay employing hemolysis of erythrocytes isolated from human blood, carried out with a PBS solution pre-incubated with the membranes for 48 h. We followed a modified protocol of Evans et al. [70]. Briefly, human blood was collected from three healthy volunteers, 6 mL of each patient’s blood was placed in a heparinized tube and centrifuged for 5 min, and then the plasma was removed. A NaCl solution of 150 mM was used to fill the tube and further centrifuged for another 5 min. The supernatant was removed and discarded. A second wash step with PBS was followed and then a solution of 1:50 erythrocytes: PBS was prepared.

To obtain a “PBS conditioned solution”, membranes were placed in a PBS solution and incubated for 48 h at 37 °C.

For hemolysis assay, 10 µL of “conditioned PBS solution” was mixed with 190 µL of erythrocytes solution and incubated for 1 h at 37 °C. The hemolysis was detected through spectrophotometric measurement of the supernatants of red blood cells treated with experimental agents at 492 nm. Additionally, as a positive control, we use 10 µL of Triton X-100 20% and a solution of 10 µL of non-conditioned PBS in 190 µL of erythrocytes solution as the negative control.

#### Cell viability assay

Cell viability was quantified using the commercial kit CellTiter 96 Aqueous One Solution Cell Proliferation Assay^®^ (Promega). Following the trader’s instructions. For this assay a tetrazolium salt, named MTS [3-(4,5-dimethylthiazol-2-yl)-5-(3-carboxymethonyphenol)-2-(4-sulfophenyl)-2Htetrazolium] is used. The MTS is reduced by cells to formazan, a colored product soluble in an aqueous solution, in a culture medium. This conversion is carried out by mitochondrial dehydrogenase enzymes present in viable cells in the culture. Here, the amount of product formed (formazan), determined from the absorbance at 490 nm is directly proportional to the number of viable cells in the culture.

To determine the cell viability of MSC attached on the membranes, we perform the assays using hBFP-MSCs. 4000 cells were seeded in each of the 96 well-plate, were previously placed the different composite membranes and cultured with α-MEM + 10% SFB for 44 h before. After that, 20 µL of culture medium was replaced with 20 µL of CellTiter 96 Aqueous One Solution Cell Proliferation Assay^®^ reagent in each well and left 4 h under incubation. The medium was removed, and the absorbance in each well was measured in a microplate reader set to 490 nm. The amount of product formed is directly proportional to the number of viable cells in the culture. The reduction of MTS achieved by control (cells grown directly on the plate) was set at 100%, and cultivate cells over the membranes were expressed as a percentage.

#### SEM analyses

The morphological characterization of the different synthesized samples and the seeded membranes was carried out in a Scanning Electron Microscopy (SEM) FEI Inspect F50 System attached with an Energy-Dispersive Spectrometer (EDX) EDAX Apollo.

The preparation of the seeded membranes for SEM analyses was performed following Romero et al. [71] modified method. Briefly, samples were fixed using 2.5% v/v glutaraldehyde in PBS for 1 h at 4 °C, then postfixed with 1% v/v OsO4 in PBS for 1 h at 4 °C and rinsed three times with distilled water. After, seeded membranes were dehydrated with a graded series of ethanol (50, 70, 80, 90, and 100% v/v), followed by a 5 min incubation with hexamethyldisilazane (HMDS. Sigma-Aldrich^®^) and left at room temperature for 2 min to dry. The samples were carbon/Pt coated in Balzers BA 510 evaporator.

#### Statistical analysis

Each experiment was performed in triplicate. One-way analysis of variance and multifactorial test (ANOVA) with *P* < 0.05 was used to determine the significant differences from different treatments.

## Results

### Structural characterization

**Fig. 1:**
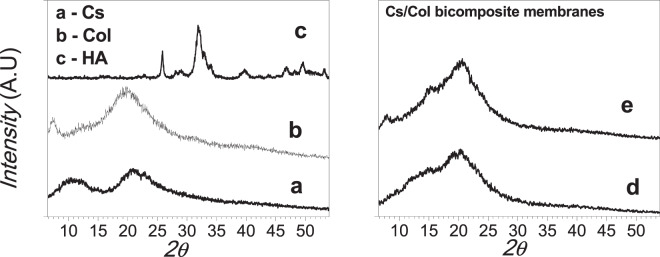
X-rays diffractograms: **a** Chitosan (Cs). **b** Collagen (Col). **c** Hydroxyapatite (HA). **d** BCC1. **e** BCC2

**Fig. 2:**
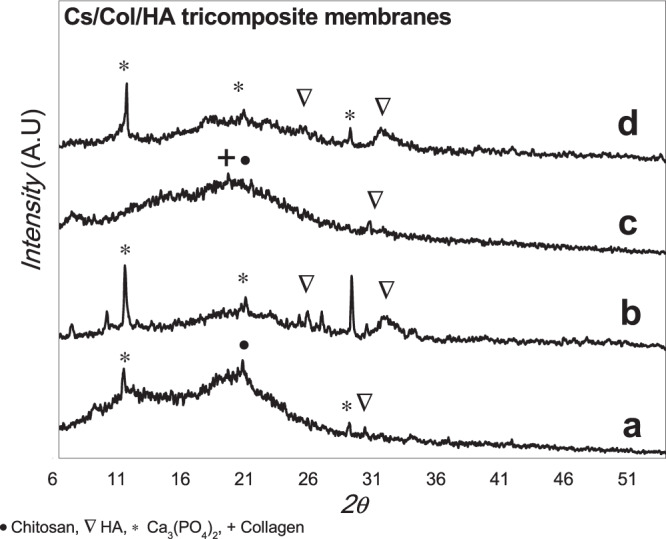
X-rays diffractograms of the different tri-composite Cs/ Col/ HA membranes: **a** TC1. **b** TC2 **c** TC3 **d** TC4

The structural analysis was carried out by XRD and FTIR analysis. The XRD patterns of the pure samples, chitosan, collagen, hydroxyapatite, and the different composites membranes are presented in Figs. 1 and 2, respectively. The XRD pattern of chitosan membrane (Fig. 1a) shows two principal peaks at 2θ = 10.87 and 20.93°, corresponding to (020) and (200) planes, respectively, characteristic of chitosan hydrate allomorph. Figure 1b shows the XRD pattern of collagen with the characteristic peaks centered at 2θ = 7.56° and 19.64° [67]. Figure 1c presents broad peaks corresponding to synthetized nanometric HA (JCPDS 9-432). The composites membranes Cs/Col0.15 (BC1) and Cs/Col0.75(BC2) are presented in Fig. 1d, e, respectively. Both materials showed a main peak around 2θ = 20.5°, corresponding to an overlap of the main reflections of chitosan and collagen. The low angle reflection characteristic of hydrated chitosan at 2θ = 10.87° has disappeared attributed to the collagen interaction with chitosan chains influencing its reorganization.

For the composites, Cs/HA, the formation of hydroxyapatite and a small amount of calcium phosphate ((Ca_3_(PO_4_)_2_ (JCPDS 006-0200)) compounds were observed.

The XRD of the chitosan/collagen/hydroxyapatite composite membranes (Cs/Col/HA) is shown in Fig. 2. For low and high HA content, the presence of hydroxyapatite, and also the formation of calcium phosphate (Ca_3_(PO_4_)_2_ (JCPDS 006-0200) was observed due to partial decomposition of HA. The acidic media partially solubilizes hydroxyapatite, and the metal ions in solution interact with chitosan resulting in the formation of calcium compounds [72, 73]. Chitosan can form complexes in solution (by the protonation of the amine groups) with different metal ions.

**Fig. 3:**
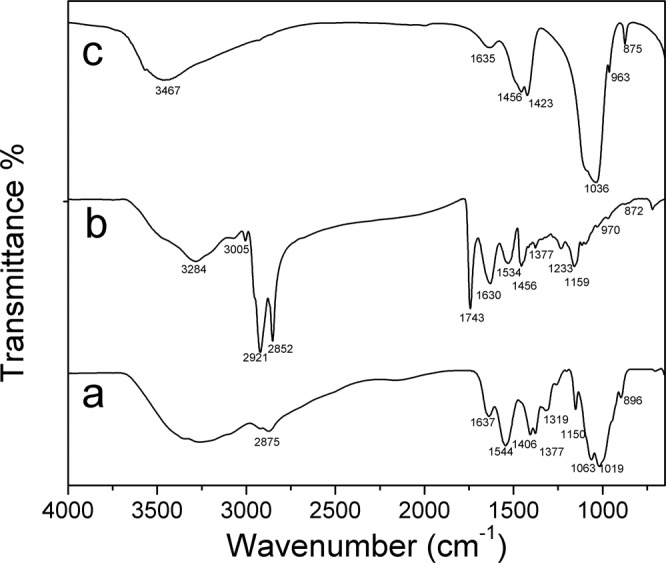
FTIR spectra: **a** Chitosan (Cs). **b** Collagen (Col). **c** Hydroxyapatite (HA)

**Fig. 4:**
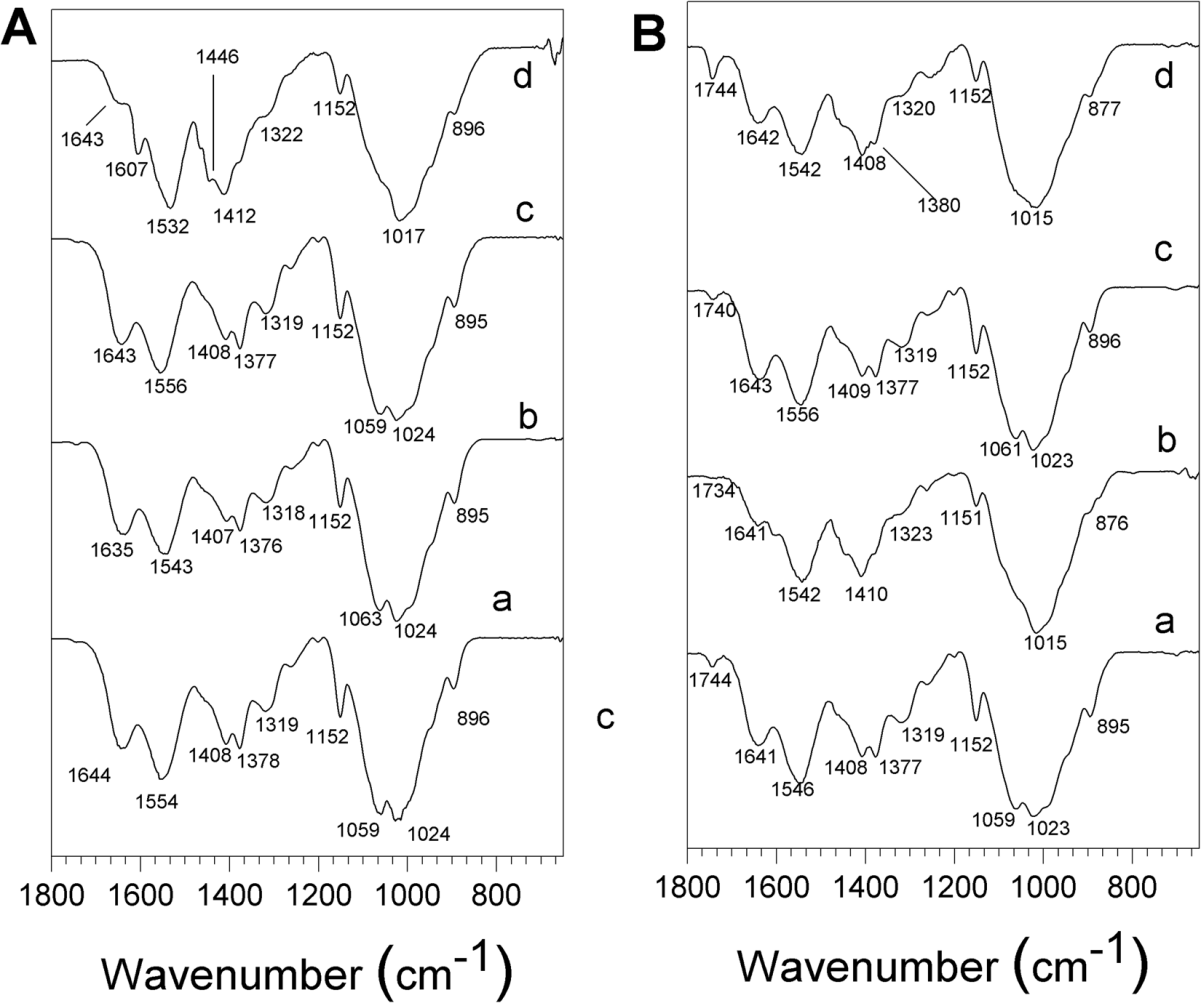
FTIR-HATR spectra of the different membranes, region 600–1800 cm^−1^ **A** composite membranes a BCC1. b BCC2. c BCHA3. d BCHA4. **B** tri-composite membranes a TC1. b TC2. **c** TC3. **d** TC4

The FTIR spectra of chitosan, collagen, hydroxyapatite are shown in Fig. 3. The characteristic absorption bands of chitosan are observed: the vibration band at 3266 cm^−1^ corresponding to the N–H stretching and –OH attributed to inter and intra-molecular hydrogen bonding, an absorption band around 2921 cm^−1^ associated to asymmetrical C–H stretch of –CH_2_ group, a vibration band at 1645 cm^−1^ assigned to C=O stretching of amide I, the N–H deformation of amide II at 1545 cm^−1^, the band corresponding to deformation C–CH_3_ at 1377 cm^−1^ and the C–O–C stretching mode characteristic of the saccharide structure at 1150, 1060, and 1020 cm^−1^. In Fig. 3b the characteristic absorption bands of collagen are observed around 3284 cm^−1^ for N–H stretching for amide I, C–H stretching at 3073 cm^−1^, a C–H vibration at 2920 cm^−1^, C=O stretching for amide I around 1743 and 1630 cm^−1^, N–H deformation at 1529 and 1233 cm^−1^, vibration bands at 1159, 970 cm^−1^ associated to C–O–C and 872 cm^−1^ associated to C–O, respectively. The FTIR spectra of HA (Fig. 4c) present the typical absorption bands, the characteristic O–H stretching modes ns at 3570 cm^−1^, the vibration band at 1036 cm^−1^ attributed to ν3 PO_4_^3−^ groups and the ν1 P–O bonds of the phosphate group at 963 cm^−1^, and the hydrogen phosphate group at 875 cm^−1^. The carbonate groups at 1418 and 1471 cm^−1^ are also present.

The enlarged region from 600 to 1800 cm^−1^ of the spectra of the different composite membranes Cs/Col, Cs/HA, and Cs/Col/HA is presented in Fig. 4. For Cs/Col the typical absorption bands of chitosan around 1645 cm^−1^ assigned to the C=O stretching of amide I remains at this frequency for low collagen content but is shifted to 1635 cm^−1^ for high collagen content, and the band at 1545 cm^−1^ corresponding to N–H deformation of amide II is shifted to 1554 cm^−1^ for low collagen content (Fig. 4a). When the collagen content increases, this band is located at 1543 cm^−1^ (Fig. 4Ab), suggesting an interaction between collagen and chitosan through the amide II groups that may occur by hydrogen bonds formation. The absorption bands of the composite membranes Cs/HA in the region 1150–1020 cm^−1^ present overlapping between the C–O–C vibration bands of chitosan with the PO_4_^3−^ vibration bands of HA. Two new absorption bands were observed at 1607 and 1446 cm^−1^ attributed to antisymmetric and symmetric stretching bands of COO– groups. Also, the band at 1545 cm^−1^ of chitosan is shifted to 1532 cm^−1^ in the Cs-Ha composite (Fig. 4b) [74]. These changes would suggest the interaction between the positively charged amino group (–NH_2_) of chitosan and PO_4_^3−^ of HA [52, 75]. The composite membranes Cs/Col/HA show all the absorption bands corresponding to chitosan, collagen, and hydroxyapatite (Fig. 6).

#### Morphological characterization

**Fig. 5:**
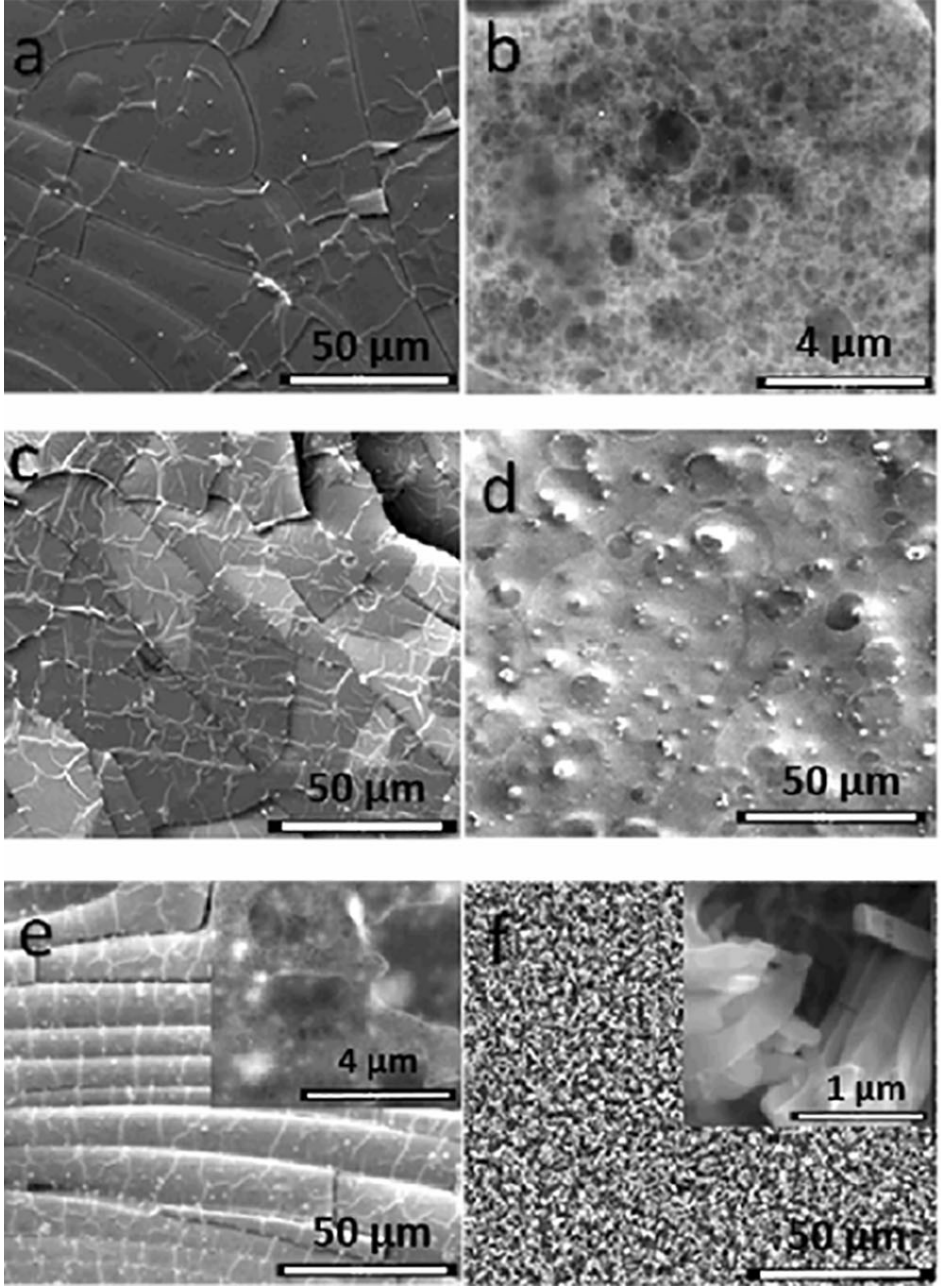
SEM Images of pure chitosan and the bi-composite Ch/Col and Ch/HA membranes **a**, **b** Chitosan (Cs). **c** BC1. **d** BC2. **e** BC3. **f** BC4; insets are magnified regions of the matrix

**Fig. 6:**
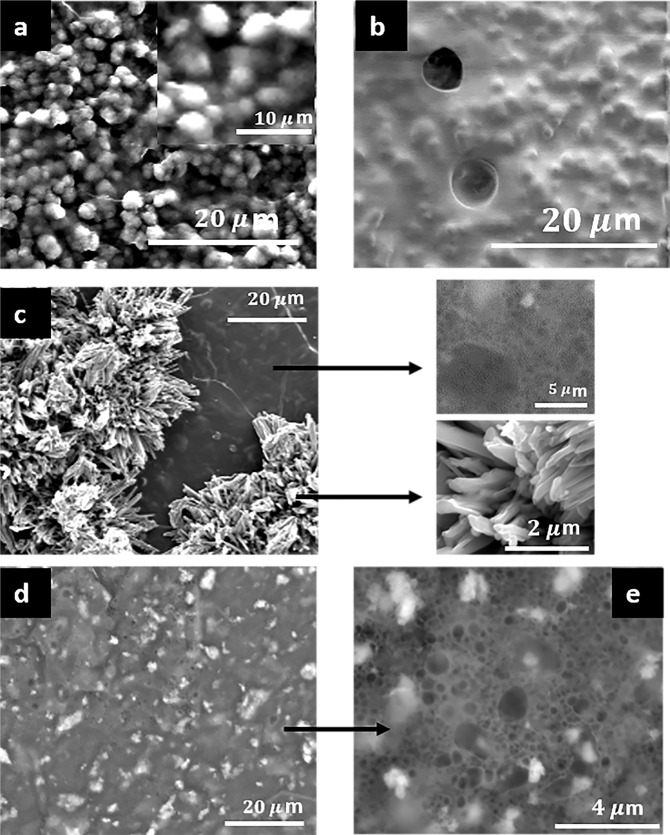
SEM Images of the different tri-composite (Cs/Col/HA) membranes: **a** TC1. **b** TC3. **c** TC2. **d**, **e** TC4. insets in **c** are magnified regions of the matrix

The morphological characterization of the different composite membranes Cs/Col, Cs/HA, and Cs/Col/HA was analysed by SEM (Figs. 5 and 6). The chitosan membrane shows an arrangement of aligned fibres (Fig. 5a). A close look at higher magnification (Fig. 5b). shows the porosity of the membrane, with pores sizes in the range of 80 nm to 2 µm.

The addition of collagen to this composite membrane changes the morphology of the original Cs membrane. For low collagen content, many pleats were observed, and areas surrounded by fibres (Fig. 5c), suggesting that collagen regions were blended into the chitosan matrix. For high collagen content, a uniform morphology was observed (Fig. 5d) characteristic of polymer blends, with regions of collagen embedded in chitosan.

The Cs/HA membranes are shown in Fig. 5e, f, for low content of HA, a good dispersion of HA was observed, with similar morphology and porosity to the pure chitosan membrane (Fig. 5e). For high HA content (HA = 0.75%), many crystallites were formed in the matrix. The elemental analyses of these crystallites showed Ca-rich particles with prismatic morphology (inset Fig. 5f) consistent with the XRD results. On the other hand, the analysis of the chitosan matrix showed high phosphorus content, suggesting phosphorylation of chitosan. The dissolution of chitosan in acetic acid is at low pH (~4.5), and therefore, partial decomposition of HA can occur, resulting in the presence of Ca^2+^ and PO_4_^3−^ ions in solution [72]. Due to the cationic nature of chitosan, interactions between PO_4_^3−^ ions and the protonated amide group can occur. The high affinity of chitosan with PO_4_^3−^ ions at low pH has been reported [50, 76].

The chitosan/collagen/hydroxyapatite composite membranes change their morphology for different collagen/HA ratios. The formation of HA clusters embedded in the matrix was observed for low collagen and HA content (Cs/Col0.15/HA0.15) TC1 membranes (Fig. 6a). This suggests a good assembly of these compounds by intermolecular interactions between them. The affinity of the HA groups with collagen on one side, and the chitosan-collagen interaction on the other, results in a homogenous composite membrane. The Energy-dispersive X-ray Spectroscopy (EDS) showed a Ca/P average ratio of 1.65, corresponding to a non-stoichiometric HA (Table 2).

In the composite chitosan/collagen/hydroxyapatite membrane, with low collagen and high HA content (Cs/Col0.15/HA0.75) TC2 membrane, selective areas with racemes of crystals (Ca/P ratio 1.67) and regions where the matrix is predominantly free of crystals are observed. The high magnification image of these regions (inset in Fig. 6c) shows a porous morphology for the matrix and the presence of mainly prismatic crystals.

For high collagen and low HA content, TC3 membrane, (Cs/Col0.75/HA0.15), HA particles are observed embedded in matrix with a good distribution (Fig. 6b). The XRD and EDS analysis were consistent with the presence of HA crystals with a Ca/P ratio 1.67. The highest content of both compounds (Cs/Col0.75/HA0.55) TC4 membrane results in a very homogeneous dispersion of HA in the porous chitosan matrix (Fig. 6d, e). The differences in morphology observed for the different membranes, with high and low collagen content, are connected to the high interaction of collagen and hydroxyapatite [77]. The membranes with high collagen (low and high HA content) present a uniform distribution of hydroxyapatite crystals in the matrix.

#### Thermogravimetric analysis (TGA)

**Fig. 7:**
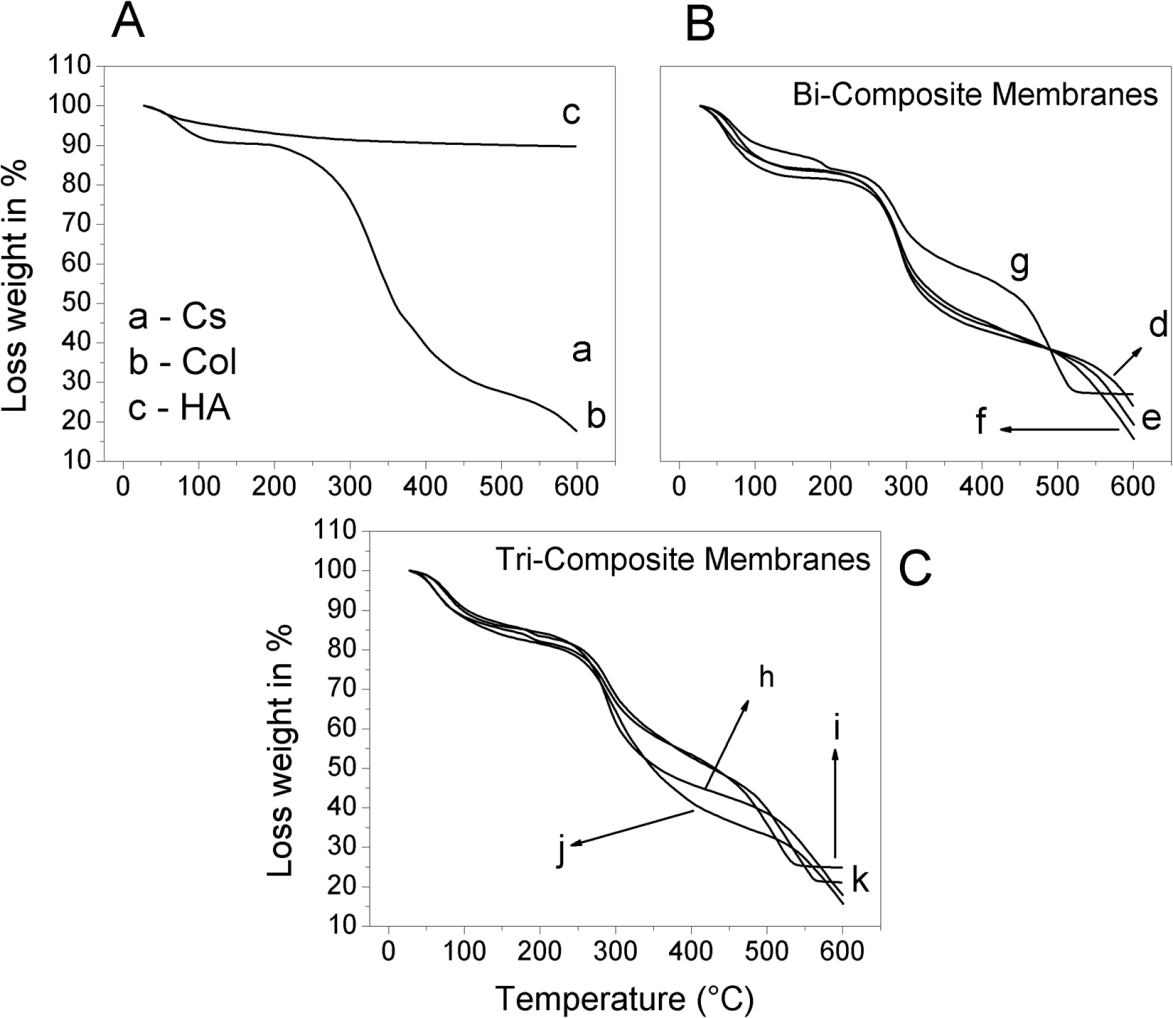
TGA analysis of the different membranes: **A** a Chitosan (Cs). b Collagen (Col). c Hydroxyapatite(HA). **B** Bi-composite d BC1. e BC2. f BC3. g BC4. **C** Tri-composite h TC1. i TC2. j TC3. k TC4

The thermal decomposition of the chitosan (Cs), Collagen (Col), Hydroxyapatite (HA), and the different composite membranes was assessed by TGA analysis. Two principal weight losses were observed for the chitosan and collagen control samples (Fig. 7A). The first round 105 °C attributed to the water evaporation present in the matrix; for the chitosan, the weight loss was near 15%, while pure collagen was near 8%. A second and drastic weight loss of around 30% was observed with the onset at 217 °C for the chitosan, attributed to the chitosan moieties due to thermal degradation. For collagen, a weight-loss onset was observed at 180 °C associated with evaporation of structural water responsible for the triple helix stability and the denaturation of dry collagen fibers, as has been reported by Bozec et al. [78]. A third transition was observed around 350 °C attributed to thermal degradation. HA presented good thermal stability in all the range of temperature-scanned showing a slight weight loss around 10% up to 600 °C. The Cs/Col composite membrane (Fig. 7B–a and b) showed the onset of thermal decomposition at 211 °C, slightly lower than for pure chitosan but higher than collagen. The weight loss was lower in this composite (23%) than for the pure elements, suggesting that the interaction of both compounds improved the thermal stability. The latter could be explained by the formation of an interconnected network between collagen and chitosan, giving better stability to the composite membrane. For the Cs/HA composite with low content of HA, important differences were not observed (Fig. 7B–c). However, increasing the HA content (0.75%), a new weight loss was present around 200 °C that might be attributed to the interaction of HA with chitosan (Fig. 7B–d). The second onset was observed at 217 °C, and a weight loss of 13% was obtained. At 300 °C a third weight loss was observed attributed to thermal decomposition of chitosan and finally a fourth weight loss with onset at 450 °C up to 519 °C due to burning out of chitosan matter. Figure 7C shows the TGA analysis of composite membranes Cs/Col/HA. In the Chitosan/HA and chitosan/collagen membranes, it was observed that collagen and HA, separately enhance the thermal stability of the chitosan matrix. The blending of both collagen and HA in chitosan further enhances the thermal properties of the chitosan composite).

**Table 2:** EDS analysis for different tri-composite membranes

Material	Ca(K) [% masse]	P(K) [% masse]	Ca/P
TC1	5.38	3.25	1.65
TC2	5.72	3.42	1.67
TC3	4.58	2.78	1.65
TC4	5.84	3.49	1.67

The higher thermal stability was obtained with high HA content; in these membranes, decomposition at 200 °C was observed attributed to the interaction of HA with the organic matrix. The third onset of weight loss was found at 217 °C, and for these composites (Cs/Col/HA) with high HA, a weight loss of 17% was observed. For the composites with low HA content, the thermal stability was slightly lower, and the third onset was observed at 205 °C with a weight loss of 40% for high collagen and 34% for low collagen. A shifting of the final decomposition temperature was observed with collagen content, from 519 °C for the composite BC4 (Cs/HA0.75) to 535 °C for the TC1(Cs/Col0.15/HA0.75), and 562 °C for high collagen content membranes TC4(Cs/Col0.75/HA0.75). While for composites Cs/Col/HA membranes with low HA content, the final decomposition temperature was 450 °C, and with high HA content was 550 °C. From these results, it might be concluded the incorporating collagen and/or hydroxyapatite to chitosan membranes (Fig. 7B, C), improves the thermal decomposition stability, and the stability increases with increasing HA content. In good agreement with studies on the evaluation of 3D scaffolds [46], these authors reported that the addition of collagen to chitosan/HA scaffolds provides thermal stability due to the covalent interactions between chitosan, HA, and collagen.

#### Cytotoxicity assay

To determine if these materials can be used for further viability assays, we first assess cytotoxicity using the hemolysis test because it was effective and provided enough information to decide if we begin the cell viability tests [69, 79, 80].

A hemolysis test was carried out with PBS in contact with the membranes for 48 h (*N* = 3). A hemolysis rate lower than 5% was obtained for all cases tested. Although there was no significant difference, the membrane with the lowest hydroxyapatite content showed a slightly higher hemolysis rate (Table 3). Therefore, it can be concluded that none of the membranes delivers toxic compounds; this is in agreement with the results of several reports [29]. Meaning, these membranes have a very low hemolytic index and can be applied for much deeper biocompatibility tests to establish if they could be used in tissue engineering.

**Table 3:** Hemolysis test results

	Cs	BC1	BC2	BC3	BC4	TC1	TC2	TC3	TC4
%Hemolysis									
± Standard deviation	0.00 ± 0.00	1.36 ± 0.34	0.25 ± 0.44	0.38 ± 0.19	0.00 ± 0.00	0.11 ± 0.00	0.27 ± 0.23	0.00 ± 0.00	0.00 ± 0.00

#### Cell viability

**Fig. 8:**
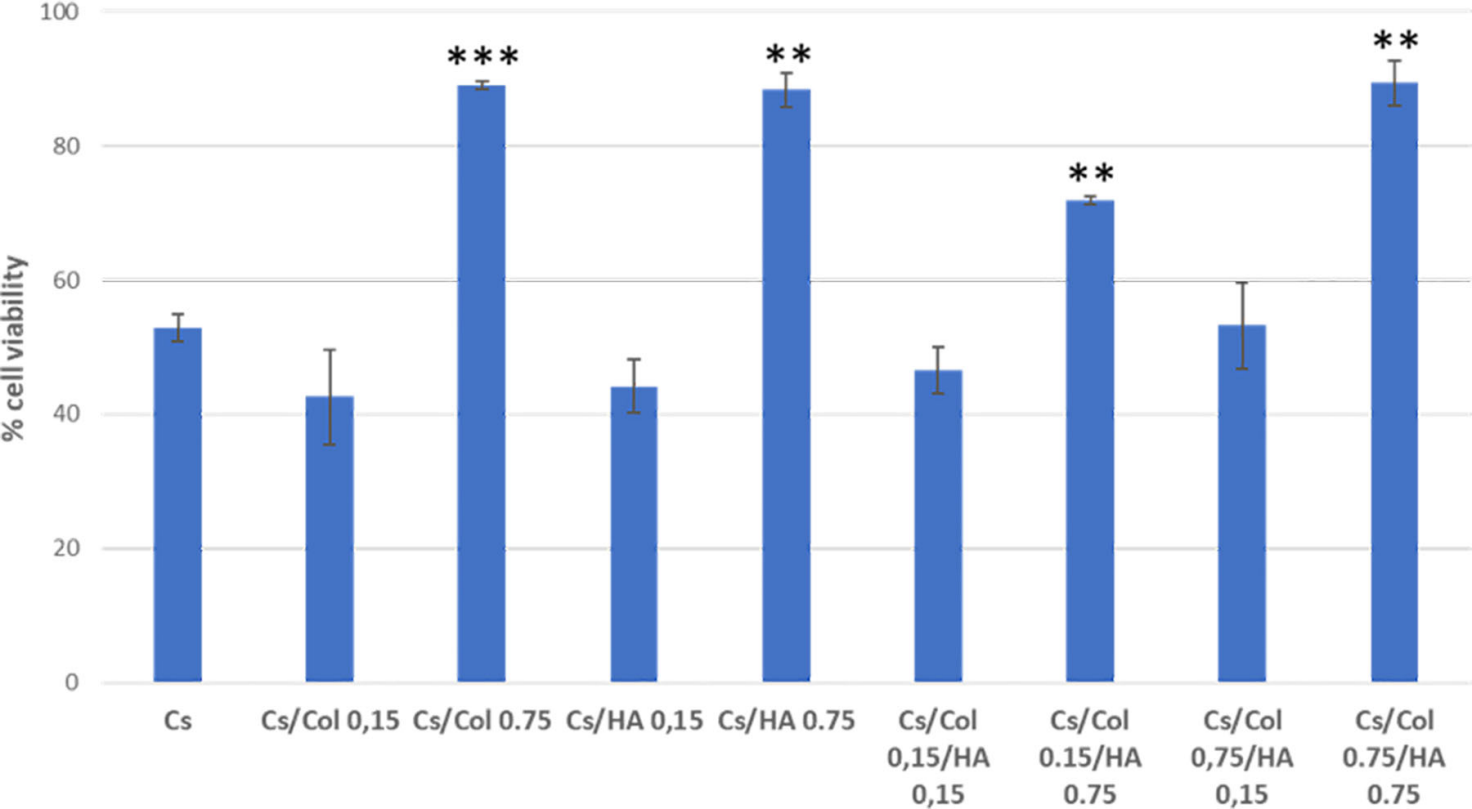
Cell viability percentage of human buccal fat pad mesenchymal stromal cells (hBFP-MSCs). Significant value is represented as *p < 0.05, **p < 0.01, ***p < 0.005 comparing with the Cs membrane (control). *N* = 6

The determination of cell viability and cell adhesion are assays of great significance since it measures in vitro the first interaction between the cell and the material’s surface, as the first step to evaluate the potential of biocompatibility of the specific material. The concept should not be confused with toxicity, which refers to the potential harm that a biomaterial may cause l, while biocompatibility refers to the performance of the materials in the physiological medium [37–39]. In the present work, we evaluated cell viability by MTS after 48 h of culture; Fig. 8 shows the results for hBFP-MSCs. Cell viability is an indirect measure of cell adhesion.

As can be observed, the membranes with the highest content of collagen and/or hydroxyapatite show the highest values of cell adhesion for in vitro cultured hBFP-MSC. The membranes with low collagen and/or low hydroxyapatite content showed similar cell viability to the pure chitosan membrane.

The largest cell viability was obtained when both compounds were present in the chitosan membrane (TC4) with the highest content of collagen and hydroxyapatite (Cs/0.75collagen/0.75 hydroxyapatite), showing 75% higher viability than the pure chitosan membrane. The latter can be associated with the high biocompatibility of collagen and hydroxyapatite and their good and uniform dispersion in the matrix (Fig. 6d, e). Moreover, the high collagen content membrane inhibits the decomposition of HA, due to the strong interaction between collagen and hydroxyapatite. These results suggest that these materials have a significant potential to be used as biomaterials for bone tissue engineering.

**Fig. 9:**
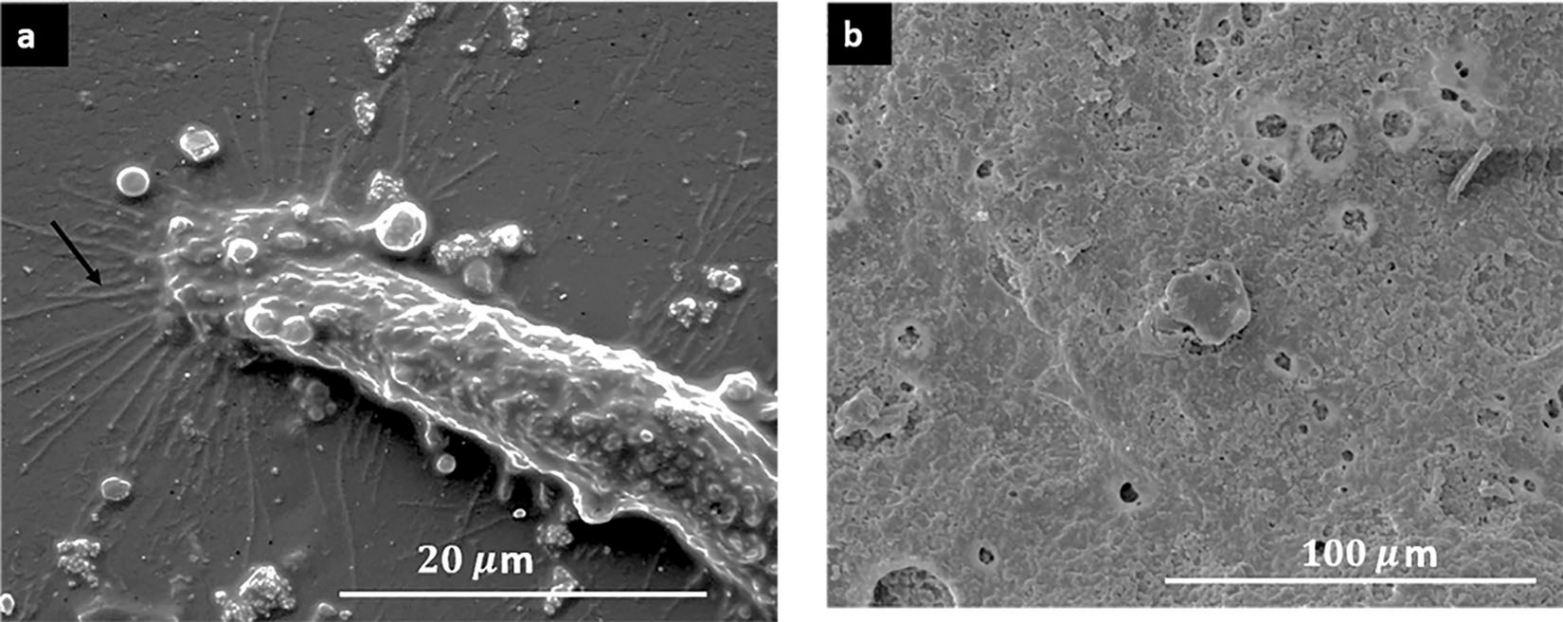
SEM Images of chitosan/collagen/hydroxyapatite (Cs/Col/HA) membranes seeded with hBFP-MSCs. **a** Chitosan (Cs). **b** TC4 (Cs/Col 0.75/HA 0.15) (*N* = 2)

Figure 9 shows the presence of filopodia indicating MSC attached to the membrane surface. In the membranes with high collagen concentration, with and without HA, the MSC were attached and proliferated in the cavities left by solubilized collagen by biological processes or culture medium. Overall, the SEM images confirm the cell adhesion indirectly quantified by the MTS assay. In the membranes with the highest viability, it was not possible to observe the formation of filopodia (Fig. 9b), due to the high confluency of hBFP-MSCs attached to the membrane.

## Discussion

The study’s main objective was the preparation and characterization of chitosan-based composite membranes, incorporating collagen and/or hydroxyapatite in different proportions for potential uses in different tissue engineering applications. Biocomposite and tri-composite membranes Cs/Col, Cs/Ha, and Cs/Col/Ha were studied.

The interaction between chitosan and collagen chains has been demonstrated by the different characterization techniques employed. The XRD showed that the characteristic low angle reflection of hydrated chitosan at 2θ = 10.87° is no present in the Col/Cs membranes, suggesting a reorganization of the molecules in these composites. This is consistent with the shifting observed by FTIR in the amide II groups, which increases with increasing collagen content, suggesting intermolecular interactions by hydrogen bonds formed between collagen and chitosan through these groups, as reported by different authors [81–85]. Sionkowska et al. [85] suggests the formation of a new compound with an entanglement of the two macromolecules forming complexes between the cationic groups of chitosan and the anionic –COOH groups of collagen, instead of forming a simple biphasic mixture. Our results are in agreement with this proposal. Also, denaturation of collagen was not observed since if that were the case, a shift in the peak at 1560 cm^−1^ would be expected [86].

The changes in morphology observed by SEM with the addition of collagen to chitosan are consistent with these results. For low collagen content, many pleats were observed, and areas surrounded by fibers (Fig. 5c), suggesting that collagen regions were blended into the chitosan matrix. For high collagen content, a uniform morphology was observed (Fig. 5d) with regions of collagen embedded in chitosan. Additionally, the weight loss observed by TGA suggests the interaction between both compounds that improve its thermal stability through the formation of an interconnected network stabilized by electrostatic interactions.

For Cs/HA composites, the FTIR analysis showed two new absorption bands at 1607 and 1446 cm^−1^, attributed to antisymmetric and symmetric stretching bands of COO– groups, and the band at 1545 cm^−1^ of chitosan shifted to 1532 cm^−1^ (Fig. 4b) [74], this suggests the interaction between the positively charged amino group (–NH_2_) of chitosan and PO43– of HA [52, 75]. This behavior is consistent with SEM’s morphological characterization, which showed well distributed crystals in the matrix. Moreover, the EDS analysis showed high phosphorus content in the chitosan matrix of these composite membranes, suggesting phosphorylation of chitosan due to the partial decomposition of HA in acid medium resulting in the presence of Ca^2+^ and PO_4_^3−^ ions in solution [72, 73]. Interaction between PO_4_^3−^ ions and the protonated amide groups of chitosan takes place [45]. The interactions between HA and chitosan were also evidenced in TGA by a weight loss around 200 °C, attributed to the interaction of calcium and phosphate ions of HA with chitosan (Fig. 7B–d). Additionally, the interaction between collagen and hydroxyapatite has been attributed to the strong electrostatic interactions between the carbonyl C=O group of collagen and the Ca ion on the hydroxyapatite surface [83–87].

**Fig. 10:**
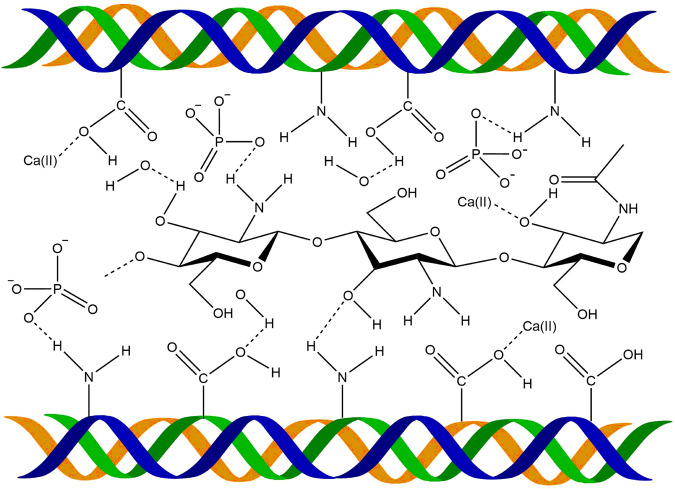
Scheme of interactions collagen/chitosan/hydroxyapatite

The FTIR for the TC membranes Cs/Col/HA (Fig. 6) showed a shift of the amide II vibration band at 1554–1546 cm^−1^ with the addition of hydroxyapatite. The latter is attributed to the intermolecular interaction between the amino groups of chitosan/collagen system and the OH, Ca+, and phosphate groups of hydroxyapatite [88]. A scheme of the interactions collagen/chitosan/hydroxyapatite is proposed below (Fig. 10).

The XRD for the TC membranes showed hydroxyapatite’s presence and the formation of a calcium phosphate due to partial decomposition of HA in acidic media [72]. The morphology of the TC membranes changes for the different combinations of low and high collagen and HA content. For low collagen content (TC1 and TC2), HA clusters and large crystals embedded in the matrix were formed. However, when the collagen content was high (TC3 and TC4) a homogenous distribution of HA crystals in a porous matrix was observed (Fig. 6b, d, e), suggesting a good assembly of these compounds by intermolecular interactions between them. The affinity of the HA groups with collagen on one side and the chitosan-collagen interaction on the other results in a homogenous composite membrane with very good dispersion of hydroxyapatite nanocrystals with Ca/P ratio 1.67.

Incorporating collagen and/or hydroxyapatite to chitosan enhances the thermal stability of chitosan (Fig. 7B, C), increasing with increased HA content, in good agreement with reports on the evaluation of 3D scaffolds [46]. The latter is attributed to the interaction between NH2 groups of chitosan and OH groups of HA and the interaction of the collagen molecules with HA particles.

For biocompatibility and viability assays, we select carefully two assays, first a hemolysis test, to quickly assess if these membranes can be used in further biocompatibility assays, followed by a MTS assay to determine if hBFP-MSC survive and attach to these membranes. The main reason to selected these assays was to determine these parameters without destroying the membrane or altering the interaction between cells and membranes.

We can conclude from the hemolysis assay that these materials do not produce soluble cytotoxic substances since the hemolysis rate was less than 5% [89] and therefore can be analyzed further to assess cell viability. To confirm the cell adhesion to the membranes, we used SEM. Regarding the results of the MTS assay, we used two cell types with adherent nature, which means that if there were no early adhesion between the first culture hours, an apoptotic signal path would be activated, decreasing the viability [90]. Therefore, the percentage of cells detected in the MTS assay attached to the material can be considered viable cells. Allowing to affirm that, in general, the membrane’s surface promotes high cell adhesion, particularly membranes with the highest content of collagen and hydroxyapatite (Cs/HA 0.75/Col 0.75). This can be associated with the high biocompatibility of collagen and hydroxyapatite and the very homogeneous HA dispersion in the porous matrix (Fig. 6d, e). The SEM confirmed that cells were well attached to the membranes. These results were similar to other publications that used chitosan combined with collagen for myocardial infarction. In these hydrogels, cells were attached and maintained high viability [91]. Other authors proposed using chitosan combined with hydroxyapatite and showed very high biocompatibility [92]. We propose that chitosan combined with hydroxyapatite and collagen promotes cell viability of culture cells attached to these membranes.

Considering these preliminary results from biocompatibility, cell viability, and cell adhesion, we could start to make more specific assays to determine if these membranes can be used in specific applications such as bone or skin tissue engineering.

Although the combination of hydroxyapatite with collagen and chitosan has been mainly focussed on developing implants that promote the repair or regeneration of bone tissue, however, studies with fibroblasts demonstrated that hydroxyapatite could stimulate the production of tissue’s collagen [10] and combinations of hydroxyapatite with other compounds have been successfully used for wound dressing [11].

In the present work, we demonstrated that the membranes obtained by combining chitosan with collagen and hydroxyapatite enhance cell adhesion ability, compared to the chitosan membranes alone, and the content of collagen and HA plays an important role. Membranes with high collagen or/and hydroxyapatite content result in better physico-chemical properties and larger cell adhesion due to the more homogeneous distribution of HA and the porous morphology of the matrix. Additionally, it was demonstrated that any of the composition present cytotoxicity. All the compositions stimulate cell adhesion and proliferation, presenting a high potential for application in tissue engineering.

## Conclusions

Composite membranes of chitosan/collagen, chitosan/hydroxyapatite, and chitosan/collagen/hydroxyapatite were successfully prepared by solvent casting method. Membranes with micro and nanopores were obtained with good dispersion of hydroxyapatite in the organic matrix. The addition of collagen and hydroxyapatite to chitosan improves thermal stability and reduces thermal decomposition of the composites. The membranes with the highest hydroxyapatite and collagen content showed the highest cell adhesion, and no cytotoxicity was presented by any of the membranes prepared, suggesting that these materials have a significant potential to be used for tissue engineering application.

## References

[1] Persidis A (1999). Tissue engineering. Nat Biotechnol.

[2] Amini AR, Laurencin CT, Nukavarapu SP, Bone Tissue Engineering: Recent Advances and Challenges. 2013;40:363–408. 10.1615/CritRevBiomedEng.v40.i5.10. 10.1615/critrevbiomedeng.v40.i5.10 PMC3766369 23339648

[3] Freedman BR, Mooney DJ (2019). Biomaterials to mimic and heal connective tissues. Adv Mater.

[4] Trevisol TC, Langbehn RK, Battiston S, Immich APS (2019). Nonwoven membranes for tissue engineering: an overview of cartilage, epithelium, and bone regeneration. J Biomater Sci Polym Ed.

[5] Baranwal A, Kumar A, Priyadharshini A, Ogg GS, Bhatnagar I, Srivastava A (2018). Chitosan: an undisputed bio-fabrication material for tissue engineering and bio-sensing applications. Int J Biol Macromol.

[6] Suchanek W, Yoshimura M (1998). Processing and properties of hydroxyapatite-based biomaterials for use as hard tissue replacement implants. J Mater Res.

[7] Pandey A, Midha S, Kumar R, Maurya R, Kumar V (2018). Antioxidant and antibacterial hydroxyapatite-based biocomposite for orthopedic applications. Mater Sci Eng C.

[8] Kalantari E, Naghib SM, Iravani NJ, Esmaeili R, Naimi-Jamal MR, Mozafari M. Biocomposites based on hydroxyapatite matrix reinforced with nanostructured monticellite (CaMgSiO_4_) for biomedical applications: Synthesis, characterization and biological studies. Mater Sci Eng C. 2019;109912. 10.1016/j.msec.2019.109912. 10.1016/j.msec.2019.109912 31546348

[9] Ramana Ramya J, Thanigai Arul K, Sathiamurthi P, Asokan K (2016). S. Narayana Kalkura, Novel gamma-irradiated agarose-gelatin-hydroxyapatite nanocomposite scaffolds for skin tissue regeneration. Ceram Int..

[10] Zerbinati N, Rauso R, Gonzalez P, Cherubino M, D’Este E, Calligaro A (2017). In vitro evaluation of collagen production of human fibroblast treated with hyaluronic acid PEG cross-linked with micromolecules of calcium hydroxyapatite in low concentration. J Biol Regul Homeost Agents..

[11] Okabayashi R, Nakamura M, Okabayashi T, Tanaka Y, Nagai A. Efficacy of Polarized Hydroxyapatite and Silk Fibroin Composite Dressing Gel on Epidermal Recovery From Full-Thickness Skin Wounds. 2009;641–6. 10.1002/jbm.b.31329. 10.1002/jbm.b.31329 19213051

[12] Geese K (2003). Collagens—structure, function, and biosynthesis. Adv Drug Deliv Rev.

[13] Tal H, Moses O, Kozlovsky A, Nemcovsky C. Bioresorbable Collagen Membranes for Guided Bone Regeneration, In: Bone Regen. 2012. 10.5772/34667.

[14] Ferreira AM, Gentile P, Chiono V, Ciardelli G (2012). Collagen for bone tissue regeneration. Acta Biomater.

[15] Parenteau-Bareil R, Gauvin R, Berthod F (2010). Collagen-based biomaterials for tissue engineering applications. Materials.

[16] Sela MN, Babinski E, Steinberg D, Kohavi D, Rosen G (2009). Degradation of collagen-guided tissue regeneration membranes by proteolytic enzymes of Porphyromonas gingivalis and its inhibition by antibacterial agents. Clin Oral Implants Res.

[17] Aurora A, Jorgic-Srdjak K. Membranes for periodontal. Regeneration, Acta Stomat Croat. 2005;39:107–12. https://hrcak.srce.hr/896.

[18] Kew SJ, Gwynne JH, Enea D, Abu-Rub M, Pandit A, Zeugolis D (2011). Regeneration and repair of tendon and ligament tissue using collagen fiber biomaterials. Acta Biomater.

[19] Quade M, Schumacher M, Bernhardt A, Lode A, Kampschulte M, Voß A (2017). Strontium-modi fi cation of porous sca ff olds from mineralized collagen for potential use in bone defect therapy. Mater Sci Eng C.

[20] Nudelman F, Lausch AJ, Sommerdijk NAJM, Sone ED (2013). In vitro models of collagen biomineralization. J Struct Biol.

[21] Stevens MM (2008). Biomaterials for bone tissue engineering. Mater Today.

[22] Chen Q, Zhu C, Thomas GA (2012). Progress and challenges in biomaterials used for bone tissue engineering: bioactive glasses and elastomeric composites. Prog Biomater.

[23] McCullough M, Gomes M, Sankar J, Bhattarai N. Development of chitosan based scaffolds for Bone Regeneration: A Preliminary Report. Corpus ID: 212555531. 2017;1:15–25.

[24] Saravanan S, Chawla A, Vairamani M, Sastry TP, Subramanian KS, Selvamurugan N (2017). Scaffolds containing chitosan, gelatin and graphene oxide for bone tissue regeneration in vitro and in vivo. Int J Biol Macromol.

[25] Hermenean A, Codreanu A, Herman H, Balta C, Rosu M, Mihali CV (2017). Chitosan-Graphene Oxide 3D scaffolds as Promising Tools for Bone Regeneration in Critical-Size Mouse Calvarial Defects. Sci Rep..

[26] Du C, Cui FZ, Zhu XD, De Groot K (1999). Three-dimensional nano-HAp/collagen matrix loading with osteogenic cells in organ culture. J Biomed Mater Res.

[27] Islam MM, Shahruzzaman M, Biswas S, Nurus Sakib M, Rashid TU (2020). Chitosan-based bioactive materials in tissue engineering applications-A review. Bioact Mater.

[28] Myhr M, Hjerde R. In vitro degradation rates of partially N-acetylated chitosans in human serum. Carbohydr Res. 1997;299:99–101. http://www.sciencedirect.com/science/article/pii/S0008621596003321. Accessed 12 Nov 2011. 10.1016/s0008-6215(96)00332-1 9129298

[29] Chatelet C, Damour O, Donard A (2001). Influence of the degree of acetylation on some biological properties of chitosan films. Biomaterials.

[30] Costa-Pinto A, Reis R, Neves N (2011). Scaffolds based bone tissue engineering: the role of chitosan. Tissue Eng Part B Rev.

[31] Becerra J, Sudre G, Royaud I, Montserrat R, Verrier B, Rochas C (2017). Tuning the hydrophilic/hydrophobic balance to control the structure of chitosan films and their protein release behavior. AAPS PharmSciTech.

[32] Xu Y, Han J, Chai Y, Yuan S, Lin H, Zhang X (2018). Development of porous chitosan/tripolyphosphate scaffolds with tunable uncross-linking primary amine content for bone tissue engineering. Mater Sci Eng C.

[33] Vukajlovic D, Parker J, Bretcanu O, Novakovic K (2019). Chitosan-based polymer/bioglass composites for tissue engineering applications. Mater Sci Eng C.

[34] Kouser R, Vashist A, Rizvi MA, Ahmad S (2018). Biocompatible and mechanically robust nanocomposite hydrogels for potential applications in tissue engineering. Mater Sci Eng C.

[35] Michalska-sionkowska M, Kaczmarek B, Walczak M, Sionkowska A (2018). Antimicrobial activity of new materials based on the blends of collagen / chitosan / hyaluronic acid with gentamicin sulfate addition. Mater Sci Eng C.

[36] Francis Suh JK, Matthew HWT (2000). Application of chitosan-based polysaccharide biomaterials in cartilage tissue engineering: a review. Biomaterials.

[37] Elango J, Saravanakumar K, Rahman SU, Henrotin Y, Regenstein JM, Wu, W. et al. Chitosan-collagen 3d matrix mimics trabecular bone and regulates rankl-mediated paracrine cues of differentiated osteoblast and mesenchymal stem cells for bone marrow macrophage-derived osteoclastogenesis, Biomolecules. 2019;9. 10.3390/biom9050173. 10.3390/biom9050173 PMC6571924 31060346

[38] Ma L, Gao C, Mao Z, Zhou J, Shen J, Hu X (2003). Collagen/chitosan porous scaffolds with improved biostability for skin tissue engineering. Biomaterials.

[39] Yan LP, Wang YJ, Ren L, Wu G, Caridade SG, Fan JB (2010). Genipin-cross-linked collagen/chitosan biomimetic scaffolds for articular cartilage tissue engineering applications. J Biomed Mater Res Part A..

[40] Minabe M, Sugaya A, Satou H, Tamura T, Ogawa Y, Hori T (1988). Histological STudy of the Hydroxyapatite-collagen Complex Implants in Periodontal Osseous Defects in Dogs. J Periodontol..

[41] Yunoki S, Ikoma T, Morikawa A, Ohta K, Kikuchi M, Marukawa E, et al. Fabrication of three-dimensional porous hydroxyapatite/collagen composite with rubber-like elasticity, Mater Sci Eng C. 2007. 10.1016/j.msec.2006.11.011.

[42] A Lamarque G, Cretenet M, Viton C, Donard (2005). New route of deacetylation of chitins by means of freeze-pump out-thaw cycles. Biomacromolecules..

[43] Lamarque G, Viton C, Donard A (2004). Comparative study of the first heterogeneous deacetylation of α- and β-chitins in a multistep process. Biomacromolecules.

[44] Jeuniaux J, Voss-Foucard C, Poulicek MF, Bussers A. Sources of chitin, estimated from new data on chitin biomass production, In: S Skjak-Braek G, A.T., editor. Chitin and Chitosan. London: 1989. pp. 3–11. http://hdl.handle.net/2268/190155.

[45] Tanase CE, Popa MI, Verestiuc L (2012). Biomimetic chitosan-calcium phosphate composites with potential applications as bone substitutes: preparation and characterization. J Biomed Mater Res B Appl Biomater.

[46] Pallela R, Venkatesan J, Janapala VR, Kim S-K. Biophysicochemical evaluation of chitosan-hydroxyapatite-marine sponge collagen composite for bone tissue engineering. J Biomed Mater Res A. 2011;486–95. 10.1002/jbm.a.33292. 10.1002/jbm.a.33292 22125128

[47] Danilchenko SN (2009). Chitosan–hydroxyapatite composite biomaterials made by a one step co-precipitation method: preparation, characterization and in vivo tests. J Biol Phys Chem..

[48] Zhang J, Liu G, Wu Q, Zuo J, Qin Y, Wang J (2012). Novel mesoporous hydroxyapatite/chitosan composite for bone repair. J Bionic Eng..

[49] Kumar BYS, Isloor AM, Kumar GCM (2019). Nanohydroxyapatite reinforced chitosan composite hydrogel with tunable mechanical and biological properties for cartilage regeneration. Sci Rep..

[50] Kumar R, Prakash KH, Cheang P, Gower L, a Khor K (2008). Chitosan-mediated crystallization and assembly of hydroxyapatite nanoparticles into hybrid nanostructured films. J R Soc Interface..

[51] Deen I, Pang X, Zhitomirsky I (2012). Electrophoretic deposition of composite chitosan–halloysite nanotube–hydroxyapatite films. Colloids Surf A Physicochem Eng Asp..

[52] Li X, Nan K, Shi S, Chen H (2012). Preparation and characterization of nano-hydroxyapatite/chitosan cross-linking composite membrane intended for tissue engineering. Int J Biol Macromol..

[53] Tai H-Y, Fu E, Don T-M (2012). Calcium phosphates synthesized by reverse emulsion method for the preparation of chitosan composite membranes. Carbohydr Polym..

[54] Huang D, Niu L, Li J, Du J, Wei Y, Hu Y (2018). Reinforced chitosan membranes by microspheres for guided bone regeneration. J Mech Behav Biomed Mater..

[55] Liu J, Fang Q, Yu X, Wan Y, Xiao B. Chitosan-based nanofibrous membrane unit with gradient compositional and structural features for mimicking calcified layer in osteochondral matrix, Int J Mol Sci. 2018;19. 10.3390/ijms19082330. 10.3390/ijms19082330 PMC6121876 30096842

[56] Jo YY, Oh JH. New resorbable membrane materials for guided bone regeneration, Appl Sci. 2018;8. 10.3390/app8112157.

[57] Chu C, Deng J, Sun X, Qu Y, Man Y (2017). Collagen membrane and immune response in guided bone regeneration: recent progress and perspectives. Tissue Eng Part B Rev..

[58] Li X, Wang X, Zhao T, Gao B, Miao Y, Zhang D, et al. Guided bone regeneration using chitosan/collagen membranes in dog dehiscence- type defect model, J Oral Maxillofac Surg. 2013. 10.1016/j.joms.2013.09.042. 10.1016/j.joms.2013.09.042 24438600

[59] Ma S, Ajayi A, Liu Z, Li M, Wu M, Xiao L (2016). Asymmetric collagen/chitosan membrane containing minocycline-loaded chitosan nanoparticles for guided bone regeneration. Sci Rep..

[60] Redepenning J, Venkataraman G, Chen J, Stafford N (2003). Electrochemical preparation of chitosan/hydroxyapatite composite coatings on titanium substrates. J Biomed Mater Res Part A..

[61] Sundaram G, Ramakrishnan T, Parthasarathy H, Raja M, Raj S, disease: A cross-link of sorts!, 2018;113–8. 10.4103/jisp.jisp. 10.4103/jisp.jisp_322_17 PMC5939019 29769766

[62] Rahman MS, Rana MM, Spitzhorn L-S, Akhtar N, Hasan MZ, Choudhury N (2019). Fabrication of biocompatible porous scaffolds based on hydroxyapatite/collagen/chitosan composite for restoration of defected maxillofacial mandible bone. Prog Biomater..

[63] Türk S, Altınsoy I, Efe GÇ, Ipek M, Özacar M, Bindal C (2018). 3D porous collagen/functionalized multiwalled carbon nanotube/ chitosan/hydroxyapatite composite scaffolds for bone tissue engineering. Mater Sci Eng C.

[64] Do Amaral MB, Viana RB, Viana KB, Diagone CA, Denis AB, De Guzzi Plepis AM (2020). In vitro and in vivo response of composites based on chitosan, hydroxyapatite and collagen. Acta Sci Technol..

[65] Huang Z, Feng Q, Yu B, Li S (2011). Biomimetic properties of an injectable chitosan/nano-hydroxyapatite/collagen composite. Mater Sci Eng C..

[66] Song JM, Shin SH, Kim YD, Lee JY, Baek YJ, Yoon SY (2014). Comparative study of chitosan/fibroin–hydroxyapatite and collagen membranes for guided bone regeneration in rat calvarial defects: micro-computed tomography analysis. Int J Oral Sci..

[67] Teng S-H, Lee E-J, Wang P, Shin D-S, Kim H-E (2008). Three-layered membranes of collagen/hydroxyapatite and chitosan for guided bone regeneration. J Biomed Mater Res Part B Appl Biomater..

[68] Farré-Guasch E, Martí-Pagès C, Hernández-Alfaro F, Klein-Nuland J, Casals N (2010). Buccal fat pad, an oral access source of human adipose stem cells with potential for osteochondral tissue engineering: An in vitro study. Tissue Eng Part C Methods.

[69] Gad S, Gad-McDonald S. Safety evaluation of medical devices. Biomater Med Devices Comb Prod. 2015;1–10. 10.1201/b19086-2.

[70] Evans BC, Nelson CE, Yu SS, Beavers KR, Kim AJ, Li H, et al. Ex vivo red blood cell hemolysis assay for the evaluation of pH-responsive endosomolytic agents for cytosolic delivery of biomacromolecular drugs. J Vis Exp. 2013;e50166:1–5. 10.3791/50166. 10.3791/50166 PMC3626231 23524982

[71] Romero MA, Sánchez F, Sabino MA, Rodríguez JP, González G, Noris-Suárez K (2011). Biocompatibility study on substrates fabricated for nerve guides using scanning electron microscopy and comparing two drying sample methods. Acta Microsc.

[72] Margolis HC, Moreno EC (1992). Kinetics of hydroxyapatite dissolution in acetic, lactic, and phosphoric acid solutions. Calcif Tissue Int..

[73] Dorozhkin SV (2012). Dissolution mechanism of calcium apatites in acids: a review of literature. World J Methodol..

[74] Nara M, Tanokura M (2008). Infrared spectroscopic study of the metal-coordination structures of calcium-binding proteins. Biochem Biophys Res Commun..

[75] Jin HH, Lee CH, Lee WK, Lee JK, Park HC, Yoon SY (2008). In-situ formation of the hydroxyapatite/chitosan-alginate composite scaffolds. Mater Lett..

[76] Wan Y, Creber B, Katherine, Peppley AM, Bui VT (2003). Synthesis, characterization and ionic conductive properties of phosphorylated chitosan membranes. Macromol Chem Phys..

[77] Ficai A, Andronescu E, Ghitulica C, Voicu G, Trandafir V, Manzu D (2009). Collagen/hydroxyapatite interactions in composite biomaterials. Mater Plast..

[78] Bozec L, Odlyha M (2011). Thermal denaturation studies of collagen by microthermal analysis and atomic force microscopy. Biophys J..

[79] Ratner BD, Hoffman AS, Schoen FJ, Lemons JE. Biomaterials science: an introduction to materials in medicine. 1996. 10.1016/b978-012582460-6/50002-5.

[80] Gaspar R, Duncan R (2009). Polymeric carriers: Preclinical safety and the regulatory implications for design and development of polymer therapeutics. Adv Drug Deliv Rev..

[81] Williams DF (2008). On the mechanisms of biocompatibility. Biomaterials.

[82] Lederman M (1976). Stage II Carcinoma of the Cervix. J R Soc Med..

[83] Travel MN, Donard A (1995). Collagen and its interaction with chitosan III. Influence of physicochemical characteristics of collagen. Biomaterials.

[84] Travel M (1996). Collagen and its interactions with chitosan III. Some biological and mechanical properties. Biomaterials.

[85] Sionkowska A, Wisniewski M, Skopinska J, Kennedy CJ, Wess TJ. Molecular interactions in collagen and chitosan blends, 2004;25:795–801. 10.1016/S0142-9612(03)00595-7. 10.1016/s0142-9612(03)00595-7 14609668

[86] Doyle BB, Bendit EG, Blout ER (1975). Infrared spectroscopy of collagen and collagen-like polypeptides. Biopolymers.

[87] Cutini M, Corno M, Costa D, Ugliengo P (2019). How does collagen adsorb on hydroxyapatite? insights from Ab initio simulations on a polyproline type II model. J Phys Chem C..

[88] Wang X, Wang X, Tan Y, Zhang B, Gu Z, Li X (2009). Synthesis and evaluation of collagen-chitosan- hydroxyapatite nanocomposites for bone grafting. J Biomed Mater Res Part A..

[89] Zhang K, Zhao M, Cai L, Wang Z, Sun Y, Hu Q (2010). Preparation of chitosan/hydroxyapatite guided membrane used for periodontal tissue regeneration. Chin J Polym Sci.

[90] Horwitz E, Le Blanc K, Dominici M, Mueller I, Slaper-Cortenbach I, Marini F (2005). Clarification of the nomenclature for MSC: The International Society for Cellular Therapy position statement.. Cytotherapy.

[91] Shaghiera AD, Widiyanti P, Yusuf H. Synthesis and Characterization of Injectable Hydrogels with Varying Collagen–Chitosan–Thymosin β4 Composition for myocardial infarction. Therapy J Funct Biomater. 2018;9. 10.3390/jfb9020033 10.3390/jfb9020033 PMC6023318 29710844

[92] Przekora A, Ginalska G (2016). In vitro evaluation of the risk of inflammatory response after chitosan/HA and chitosan/β-1,3-glucan/HA bone scaffold implantation. Mater Sci Eng C.

